# Effective surface oxidation of polymer replica molds for nanoimprint lithography

**DOI:** 10.1186/1556-276X-7-39

**Published:** 2012-01-05

**Authors:** Ilhwan Ryu, Dajung Hong, Sanggyu Yim

**Affiliations:** 1 Department of Chemistry, Kookmin University, Seoul, 136-702, South Korea

## Abstract

In nanoimprint lithography, a surface oxidation process is needed to produce an effective poly(dimethylsiloxane) coating that can be used as an anti-adhesive surface of template molds. However, the conventional photooxidation technique or acidic oxidative treatment cannot be easily applied to polymer molds with nanostructures since surface etching by UV radiation or strong acids significantly damages the surface nanostructures in a short space of time. In this study, we developed a basic oxidative treatment method and consequently, an effective generation of hydroxyl groups on a nanostructured surface of polymer replica molds. The surface morphologies and water contact angles of the polymer molds indicate that this new method is relatively nondestructive and more efficient than conventional oxidation treatments.

## Introduction

Recently, nanoimprint lithography [NIL] has attracted increasing attention as a facile technique for patterning polymer nanostructures [1-3]. The principle of NIL is very simple and described in detail elsewhere [1]. A hard mold with nanoscale surface-relief features is pressed onto a polymer cast at controlled temperature and pressure, which creates replica patterns on the polymer surface. Mold materials normally used for NIL include silicon, silicon dioxide, silicon nitride, or metals such as nickel, and the surface nanostructures are typically fabricated using various lithographic, electrochemical, and etching techniques [1,4-6]. While these conventional inorganic molds are thermally and mechanically stable [7], they often easily break due to their stiffness when pressed or removed. The large mismatch of thermal expansion between stiff inorganic molds and polymeric films is also problematic. For these reasons, several attempts have been made to use soft and flexible molds made from polymeric materials [8]. Various elastomeric polymers such as poly(dimethylsiloxane) [PDMS] were used for this purpose [9-11]. However, due to the innate softness of these materials with low elastic modulus, e.g., 2 to 4 MPa for PDMS, the molds tended to deform when pressure was applied, and hence, these materials were not suitable for imprinting nanoscale features. Stiffer polymeric molds with a higher mechanical strength such as urethane- [12,13] and epoxide-based [14,15] polymer molds were therefore introduced. For example, the Norland Optical Adhesives (NOA63, Norland Products, Cranbury, NJ, USA), a urethane-based UV-curable polymer, is a plausible candidate due to its good mechanical properties and high Young's modulus (approximately 1, 655 MPa) [16]. The urethane- and epoxide-based polymers, however, possess high surface energies, leading to strong adhesion of the molds to the imprinted surface. Consequently, the mold surface must be coated with an anti-adhesive layer. Recently Kim et al. introduced the PDMS coating technology onto various hard and soft molds including these stiffer polymers. The PDMS-coated molds showed good surface properties, i.e., low surface energy and low adhesion properties, like normal PDMS molds [17,18]. It was previously reported that to create a strong and highly stable PDMS coating, the oxidized polymer surface must be treated with 3-aminopropyltriethoxysilane [APTES] before PDMS deposition. The hydroxyl groups on the oxidized polymer surface can bind strongly with APTES by silanization, and subsequent PDMS deposition forms strong covalent bonds between the aminosilane (APTES)-treated surface and monoglycidyl ether-terminated PDMS through epoxy-amine chemistry [17,18]. A well-established technique for surface oxidation and consequent generation of terminal hydroxyl groups for various semiconductors is the piranha soak (sulfuric acid and hydrogen peroxide mixed solution) [1]. This approach, however, cannot be applied to polymer surfaces since most polymeric materials are highly vulnerable to strong acids. Photooxidation using UV-oxygen treatment has been reported as an alternative [17,18]. However, this approach is also destructive [19,20], and the polymer surfaces are rapidly etched before the formation of surface hydroxyl groups is optimized. In this study, we developed a relatively nondestructive oxidation approach using a mixed solution of ammonium hydroxide and hydrogen peroxide for the generation of hydroxyl groups on NOA63 polymer surfaces and compared the effectiveness of this method with that of previously reported photooxidation approaches.

## Experimental details

**Figure 1:**
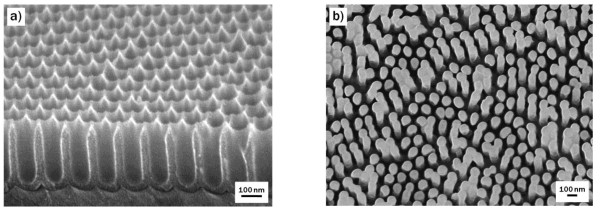
**FE-SEM images of nanoholes and nanopillars**. (**a**) Nanoholes in anodic aluminum oxide master mold and (**b**) nanopillars in NOA63 replica mold.

A nano-patterned NOA63 replica mold was fabricated using a pre-patterned anodic aluminum oxide [AAO] master mold. The ordered AAO nanohole structures (Figure 1a) with a pore diameter of approximately 65 nm and depth of approximately 220 nm were prepared via a two-step anodization process, employing 0.3 M of oxalic acid as an electrolyte at an anodization voltage of 40 V. The surface of the AAO nanoholes was then coated with an ether-terminated PDMS (*M*_n _= 5, 000; Sigma-Aldrich, St. Louis, MO, USA) layer using a coating method previously described [17] for anti-adhesion. Using the PDMS-coated AAO template as a master mold, a NOA63 polymer replica mold was prepared. The UV curable NOA63 polymer precursor was spread on the AAO mold and then pressed using a flexible polyethylene terephthalate [PET] film. After curing with UV radiation (*λ *= 365 nm) for 1 h, the replica mold was peeled off, providing a surface nanopillar-patterned NOA63 polymer layer formed on a PET film (Figure 1b). For the generation of surface hydroxyl groups, the NOA63 replica mold was oxidized using two different oxidation methods, photooxidation and basic oxidative treatment, for comparison. Photooxidation was performed using UV radiation at a peak wavelength of 254 nm and power of 15 mW/cm^2 ^(SUV110GS-36, SEN LIGHTS Corporation, Toyonaka, Osaka, Japan). For the basic oxidative treatment, the NOA63 mold was immersed into a mixture of ammonium hydroxide (25 wt.%), hydrogen peroxide (28 wt.%), and distilled water in a volumetric ratio of 1:1:5 and kept at 80°C for various periods of time. The mold was then rinsed with deionized water and blown dry with N_2 _gas. Afterward, the replica molds oxidized with both methods were immersed into a 0.5-wt.% APTES (99%; Sigma-Aldrich, St. Louis, MO, USA) aqueous solution for 10 min, which was followed by PDMS coating. The surfaces of the replica molds were analyzed *ex situ *using a field emission scanning electron microscope [FE-SEM] (JEOL JSM-7410F, JEOL Ltd., Akishima, Tokyo, Japan) and a contact angle analyzer (Phoenix 300 System, PHOENIX Restoration Equipment, Madison, WI, USA).

## Results and discussion

**Figure 2:**
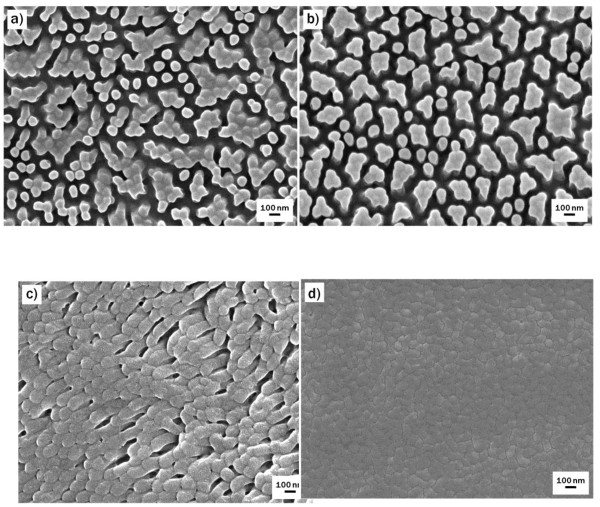
**FE-SEM images of NOA63 replica mold surfaces treated with photooxidation**. Representative FE-SEM images of NOA63 replica mold surfaces treated with photooxidation for (**a**) 1, (**b**) 2, (**c**) 4, and (**d**) 8 min.

**Figure 3:**
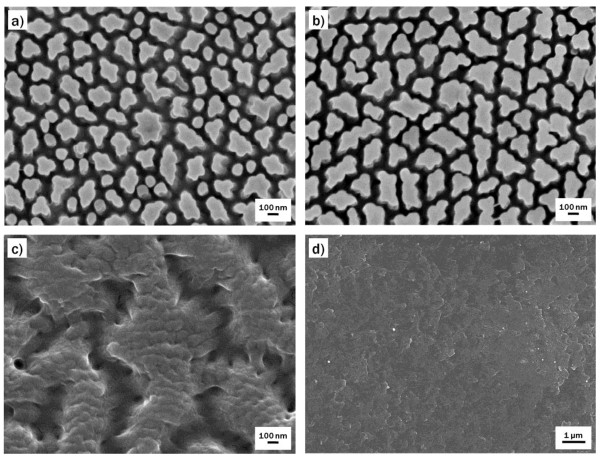
**FE-SEM images of NOA63 replica mold surfaces oxidized by basic oxidative treatment**. Representative FE-SEM images of NOA63 replica mold surfaces oxidized by basic oxidative treatment for (**a**) 5, (**b**) 10, (**c**) 15, and (**d**) 30 min.

Figure 2 shows FE-SEM images of the surface of samples treated with photooxidation for different periods of time. Short exposure to UV radiation, e.g., 1 min (Figure 2a), did not significantly alter the surface, except that a couple of neighboring nanopillars tended to adhere to one another, implying that intermolecular interactions between adjacent pillars increased as the number of surface hydroxyl groups increased. After 2 min of treatment (Figure 2b), individual nanopillars were hardly observed, and the surface was covered with irregular-shaped, agglomerated pillars that ranged in size from 80 to 350 nm. As the treatment proceeded, the surface etching as well as the generation of hydroxyl groups continued as shown in Figure 2c. The FE-SEM image indicated that the surface was entirely etched off, and its nanostructures completely disappeared after 8 min of photooxidation treatment (Figure 2d). In contrast, the surface nanostructures were retained for a relatively long time when the basic oxidative treatment was used (Figure 3). As with photooxidation, the initial basic treatment, e.g., 5 min of treatment (Figure 3a), resulted in adhesion between neighboring nanopillars. After 10 min of treatment, the whole surface was covered with agglomerated pillars that ranged from 100 to 300 nm in size (Figure 3b), which was similar to the 2-min photooxidation-treated surface (Figure 2b). FE-SEM images of the surface of the sample treated for 15 min showed that the pillar agglomeration and surface deformation continually progressed (Figure 3c), and after 30 min of treatment (Figure 3d), the nanostructures completely disappeared, as was observed after 8 min of photooxidation treatment (Figure 2d).

**Figure 4:**
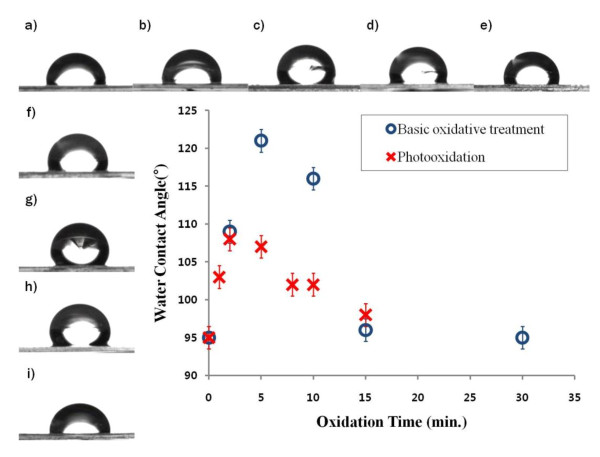
**Water contact angles of the PDMS-coated NOA63 replica molds**. Prior to silanization and PDMS coating, the molds were oxidized by UV radiation for (**a**) 0, (**b**) 1, (**c**) 2, (**d**) 5, and (**e**) 8 min and by basic oxidative treatment for (**f**) 2, (**g**) 5, (**h**) 10, and (**i**) 30 min. The contact angle values are plotted as a function of oxidation time.

The extent of the surface oxidation and hydroxyl group generation can be evaluated by measuring the water contact angle [17]. An increase in the amount of hydroxyl groups generated on the surface leads to more effective binding with APTES and subsequent PDMS and consequently produces a more hydrophobic surface. Figure 4 shows the results of the surface contact angle measurements. The measurements were carried out after APTES silanization and PDMS coating on samples oxidized by either UV radiation or basic oxidative treatment. The change in contact angle at longer oxidation times was consistent with changes in surface morphology observed by FE-SEM (Figures 2 and 3). The water contact angle of the sample not subjected to oxidation treatment was 95° ± 2° (Figure 4a). Figures 4b to 4e show the contact angles for the samples treated with UV radiation, and Figures 4f to 4i show the contact angles for the samples treated with basic oxidative solution. The angles for the samples oxidized by both treatments increased initially and then decreased at a time point when the surface was etched off. In the case of UV photooxidation, a maximum contact angle of 109° ± 2° was observed for the 2 min-treated sample. In contrast, the maximum contact angle was 121° ± 2° when the sample was oxidized for 5 min for the basic oxidative treatment. These results indicate that hydroxyl groups on nanostructured NOA63 polymer surface are more effectively generated using basic oxidative treatment than UV photooxidation. This can be explained that during UV photooxidation, there was not enough time for sufficient generation of surface hydroxyl groups due to the rapid and drastic surface etching by the UV radiation.

## Conclusions

In conclusion, a novel surface oxidation method using a basic oxidative solution was successfully developed for the generation of hydroxyl groups on a nanostructured NOA63 polymer surface. In comparison with the previously reported UV photooxidation method, this new method is relatively nondestructive and more effective based on changes in the surface morphology and contact angle.

## Competing interests

The authors declare that they have no competing interests.

## Authors' contributions

IR carried out the oxidation and data analysis. DH fabricated and provided template molds. SY designed the study and participated in the experiments. All authors read and approved the final manuscript.

## References

[B1] Guo LJ Nanoimprint lithography: methods and material requirements Adv Mater 2007 19 495

[B2] Chou SY Krauss PR Renstrom PR Imprint lithography with 25-nanometer resolution Science 1996 272 85 10.1126/science.272.5258.85

[B3] Guo LJ Recent progress in nanoimprint technology and its applications J Phys 2004 D 37 R123

[B4] Ting CJ Huang MC Tsai HY Chou CP Fu CC Low cost fabrication of the large-area anti-reflection films from polymer by nanoimprint/hot-embossing technology Nanotechnology 2008 19 205301 10.1088/0957-4484/19/20/205301 21825735

[B5] Ansari K van Kan JA Bettiol AA Watt F Fabrication of high aspect ratio 100°nm metallic stamps for nanoimprint lithography using proton beam writing Appl Phys Lett 2004 85 476 10.1063/1.1773933

[B6] Maximov I Sarwe E-L Beck M Deppert K Graczyk M Magnusson MH Montelius L Fabrication of Si-based nanoimprint stamps with sub-20 nm features Microelectron Eng 2002 61-62 449

[B7] Chou SY Krauss PR Chang W Guo L Zhuang L Sub-10 nm imprint lithography and applications J Vac Sci Technol 1997 B15 2897

[B8] Barbero DR Saifullah MSM Hoffmann P Mathieu HJ Anderson D Jones GAC Welland ME Steiner U High-resolution nanoimprinting with a robust and reusable polymer mold Adv Funct Mater 2007 17 2419 10.1002/adfm.200600710

[B9] Kim YS Suh KY Lee HH Fabrication of three-dimensional microstructures by soft molding Appl Phys Lett 2001 79 2285 10.1063/1.1407859

[B10] Narasimhan J Papautsky I Polymer embossing tools for rapid prototyping of plastic microfluidic devices J Micromech Microeng 2004 14 96 10.1088/0960-1317/14/1/013

[B11] Ge H Wu W Li Z Jung GY Olynick D Chen Y Alexander Liddle J Wang SY Williams RS Cross-linked polymer replica of a nanoimprint mold at 30 nm half-pitch Nano Lett 2005 5 179 10.1021/nl048618k 15792435

[B12] Kim YS Lee HH Hammond PT High density nanostructure transfer in soft molding using polyurethane acrylate molds and polyelectrolyte multilayers Nanotechnology 2003 14 1140 10.1088/0957-4484/14/10/312

[B13] Yoo PJ Choi S-J Kim JH Suh D Baek SJ Kim TW Lee HH Unconventional patterning with a modulus-tunable mold: from imprinting to microcontact printing Chem Mater 2004 16 5000 10.1021/cm049068u

[B14] Khang D-Y Kang H Kim T-I Lee HH Low-pressure nanoimprint lithography Nano Lett 2004 4 633 10.1021/nl049887d

[B15] Choi D-G Jeong J-H Sim Y-S Lee E-S Kim W-S Bae B-S Fluorinated organic-inorganic hybrid mold as a new stamp for nanoimprint and soft lithography Langmuir 2005 21 9390 10.1021/la0513205 16207009

[B16] Park J Kim YS Hammond PT Chemically nanopatterned surfaces using polyelectrolytes and ultraviolet-cured hard molds Nano Lett 2005 5 1347 10.1021/nl050592p 16178236

[B17] Lee MJ Lee NY Lim JR Kim JB Kim M Baik HK Kim YS Antiadhesion surface treatments of molds for high-resolution unconventional lithography Adv Mater 2006 18 3115 10.1002/adma.200601268

[B18] Kim JH Kim MH Lee MJ Lee JS Shin KS Kim YS Low-cost fabrication of transparent hard replica molds for imprinting lithography Adv Mater 2009 21 4050 10.1002/adma.200803243

[B19] Egitto FD Plasma etching and modification of organic polymers Pure & Appl Chem 1990 62 1699 10.1351/pac19906209169922229037

[B20] Grubb DT Radiation damage and electron microscopy of organic polymers J Mater Sci 1974 9 1715 10.1007/BF00540772

